# Automation of Hessian-Based Tubularity Measure Response Function in 3D Biomedical Images

**DOI:** 10.1155/2011/920401

**Published:** 2011-02-22

**Authors:** Oleksandr P. Dzyubak, Erik L. Ritman

**Affiliations:** Physiological Imaging Research Laboratory, Department of Physiology and Biomedical Engineering, Mayo Clinic, Rochester, MN 55905, USA

## Abstract

The blood vessels and nerve trees consist of tubular objects interconnected into a complex tree- or web-like structure that has a range of structural scale 5 *μ*m diameter capillaries to 3 cm aorta. This large-scale range presents two major problems; one is just making the measurements, and the other is the exponential increase of component numbers with decreasing scale. With the remarkable increase in the volume imaged by, and resolution of, modern day 3D imagers, it is almost impossible to make manual tracking of the complex multiscale parameters from those large image data sets. In addition, the manual tracking is quite subjective and unreliable. We propose a solution for automation of an adaptive nonsupervised system for tracking tubular objects based on multiscale framework and use of Hessian-based object shape detector incorporating National Library of Medicine Insight Segmentation and Registration Toolkit (ITK) image processing libraries.

## 1. Introduction

Humans (indeed all biological multicellular organisms) are made of multiscale hierarchy of structures ranging from subcellular structures (10^−7^ m) to cells (10^−5^ m), basic functional units (the smallest aggregation of diverse cells that behaves like the parent organ 10^−4^ m), organs (10^−2^–10^−1^ m), and bodies (10^0^ m). In addition to the local scale variation, biological structures are also characterized by shape. For example, the blood vessels are tubular objects interconnected into a complex network and have a range of structural scale (5 *μ*m diameter capillaries to 3 cm aorta). This large-scale range presents two major problems; one is just making the measurements, and the other is the exponential increase of component numbers with decreasing scale. Quantitative analysis of such systems, for example, blood vessel trees and networks of neurological dendrites and axons, seem to be best measured from 3D images of those structures. With the remarkable increase in the volume imaged by, and resolution of, modern day imagers, the practical problem now is the extraction of this multiscale data from those large, detailed image data sets. Thus, there is a need for an automatic tool which mines data across both space and scale to capture local information about the objects which describes the feature and now becomes associated with its appropriate position and scale.

Without prior information for a scale description of the image content, an image has to be studied at all scales. The basis for design of an automatic tool for such description could be derived from human perception model [1]. The human eye comprises a large number of individual receptors (over 150 million rods and cones). Such “imager” has no prior information about input, and therefore, it is designed to extract the information by applying sampling apertures at a wide range of sizes simultaneously [1, 2]. Since the information from a single individual sensor is almost meaningless, the sampling should be done not by the individual rods and cones in a human eye or detectors in imagers but by the sensor neighborhoods. Such sensor neighborhoods implement fundamental “multiscale perception” at different scales simultaneously but at this point no memory or analysis is involved yet. The very first level of analysis starts when grouping and storing the information from the local neighborhoods into meaningful sets (so-called “blurring signal”) and establishing interconnections between neighborhoods. Since a priori size of the features in the object (signal) is unknown, the “blurring” is used by both humans and machines for adaptive multiscale representation of meaningful signal features of a different size. Within the next step when we extract an object, certain properties have been already attributed to it, and the rest of the information is now considered as nonobject and often called “noise” or “background”. As both noise and object are always parts of the perception process, there is no way to separate the noise from the object if models of the object and of the noise are absent. To distinguish noise from object, we need to model the image content. That could be achieved by using certain mathematical operators, “feature detectors”, interacting with the data followed by analysis of the results of “feature extraction”. Since objects in images comprise features with varying size, it is quite logical to perform feature extraction at the scale which matches the local feature size, “its native scale”. A recipe for automatic feature extraction in multiscale framework can be given as follows: (i) build multiscale representation by smoothing the image at each scale; (ii) choose an appropriate “feature detector” to compute local structural properties (e.g., gradients, curvatures, flows); (iii) compute “local extrema” of a “feature detector response” function; (iv) find the strongest local response of the structural properties which is considered as feature identification. 

## 2. Background and Principle

To build the multiscale representation of the image, the proper aperture (windowing) function as an operator should be chosen. Formalism for the scale-space representation was introduced by Witkin [3] and further developed by Koenderink [4]. The idea of this approach is to generate a one parameter family of smoothed images *I*(*x*, *y*; *t*) obtained by convolving the original image *I*_0_(*x*, *y*) with a Gaussian kernel *G*(*x*, *y*; *t*) of size *t*

(1) $$\begin{array}{c} I\left(x,y;t\right)=I_{0}\left(x,y\right)*G\left(x,y;t\right),\\ \text{where}\;G\left(x,y;t\right)=\frac{1}{2\pi t}e^{-\left(x^{2}+y^{2}\right)/2t}. \end{array}$$

**Figure 1:**
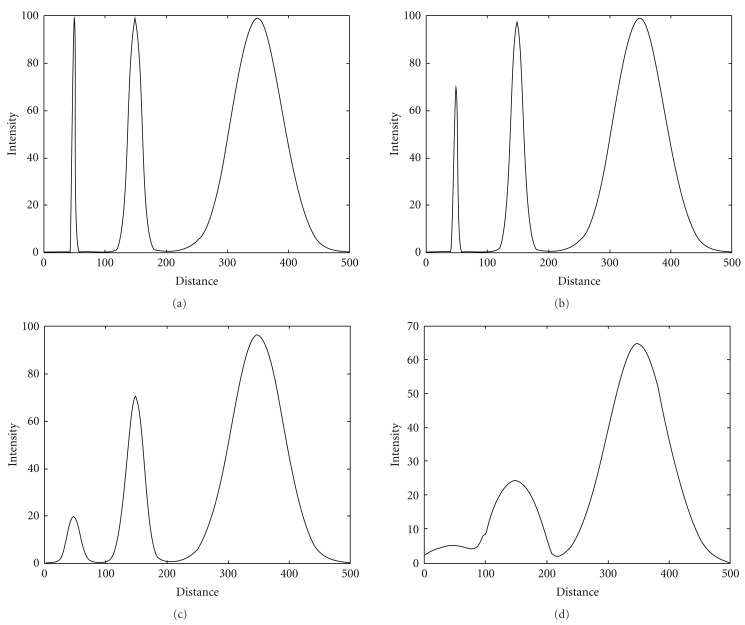
Smoothing the finer spatial image structures out with increase of Gaussian kernel size: original image with structure sizes sigma = 2; 10; 40 (a). Convolution with Gaussian using various kernel size sigma = 2 (b), 10 (c), 40 (d). With Gaussian kernel size increasing, finer image structures are disappearing.

In case of Gaussian kernels, the kernel size *t* is called variance, which is related to standard deviation *σ* as *t* = *σ*^2^. The parameter *t* in this family represents the scale at which the finer image structures are still “perceptible” whereas the spatial structures with size smaller than $\sqrt{t}=\sigma$ will be smoothed out as shown in Figure 1. As pointed out by Koenderink [4] and Hummel [5], this family of smoothed images may also be derived as the solutions of the diffusion equation

(2) $$\begin{array}{cc} \frac{\partial I\left(x,y;t\right)}{\partial t} & =\nabla \left[c\left(x,y;t\right)\nabla I\left(x,y;t\right)\right]\\ & =c\left(x,y;t\right)\Delta I\left(x,y;t\right)+\nabla c\left(x,y;t\right)\nabla I\left(x,y;t\right). \end{array}$$

If *c*(*x*, *y*; *t*) is constant, it reduces to the isotropic diffusion equation ∂*I*(*x*, *y*; *t*)/∂*t* = *c*Δ*I*(*x*, *y*; *t*), and the linear spatial scale-space representation can be generated using Gaussian (continuous) or Binomial (discrete) kernels [2–4, 6–10]. Data sampled in the temporal domain (e.g., the movie frames are samples taken at regular intervals) can also be scaled in similar to the spatial domain fashion. To treat a multiscale context over the temporal domain, it was suggested to use the Poisson kernels [11–13]. Perona and Malik [14] extended the scale-space concept to nonlinear scale-spaces based on nonlinear diffusion formulation with nonconstant diffusion coefficient *c*(*x*, *y*; *t*); (see (2)). Comprehensive overview of nonlinear scale-spaces based on parabolic partial differential equations could be found in [15, 16]. For such nonlinear cases, the Bessel scale-space can be built [17].

The scale spaces could be generated by using various kernel functions. Recently, wavelets and their applications to signal and image processing have attracted attention of the scientists in many fields. A very good collection of papers on the wavelet theory and its applications can be found in the book by Heil and Walnut [18]. The relationship between the wavelets and the scale spaces was demonstrated by Mallat and Hwang [19]. In [20–22], methods for generating scale-space representations based on wavelets were suggested, and the results of application of wavelets were very promising. As an extension of the Gaussian and wavelet approaches, Wang and Lee [23] proposed the scale-space representation derived from B-splines.

Even though different kernel functions have been proposed to generate the scale-space representations, the Gaussian kernels remain the best candidates so far [1–10]. Such “uniqueness” of the Gaussian kernels was shown by Babaud et al. [24] and is based on a priori scale-space constraint formulated by Witkin [3], Koenderink [4], and Yuille and Poggio [6]: “No new feature points (no “spurious detail”) should be created with increasing scale”. Florack et al. [25, 26] extended constraints and formulated the mathematical requirements known as “axioms for an uncommitted visual front-end” [27] which should be satisfied for the systems without any a priori knowledge about inputs: (i) *linearity*: no “feedback” from the system; (ii) *spatial shift invariance*: no preferred spatial or temporal location; (iii) *isotropy*: no preferred spatial or temporal orientation; (iv) *scale invariance*: no preferred size or scale of the aperture.

Another motivation to use the Gaussian scale spaces has some support from neurophysiological and psychological experiments which has shown that receptive field profiles in the mammalian retina and visual cortex can be well modeled by sums of Gaussian [28] and Gaussian derivative components [29–31].

All these properties make the linear Gaussian scale spaces the best choice for development of the automatic unsupervised systems for multiscale signal and image analysis, when there is no in advanced information available concerning preferable scales. The recipe which allows adaptively choosing the proper local scale parameter at every geometrical location was suggested by Lindeberg [32, 33]. This recipe comprises a two-step procedure: (i) convolving the original image *I*_0_(*x*, *y*) with a Gaussian kernel *G*(*x*, *y*; *σ*) of size *σ*; (see (1)) and (ii) analyzing a response *R*(*x*, *y*; *σ*) function from derivatives or some (possibly nonlinear) combination of derivatives of that convolution [32]

(3) $$R\left(x,y;\sigma \right)=\frac{\partial^{m+n}I\left(x,y;\sigma \right)}{\partial x^{m}\partial y^{n}},$$

where *m* and *n* are orders of derivatives.

**Figure 2:**
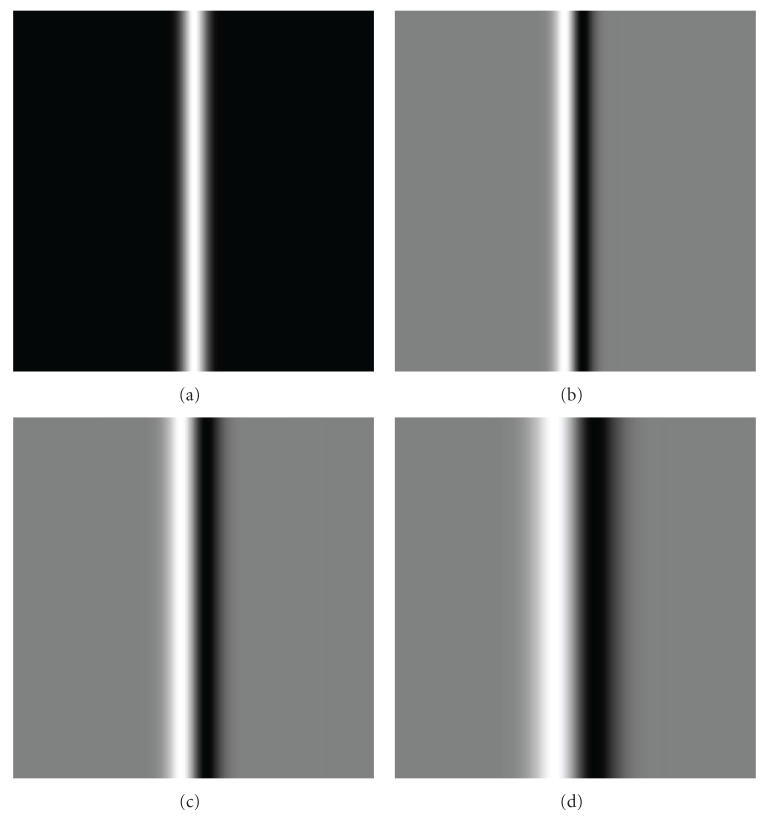
Original image convolved with first-order Gaussian derivatives. (a) Original image having an object with Gaussian intensity profile of kernel size *σ* = 5 (a). (b) Original image convolved with first order Gaussian Derivative of kernel size *σ* = 1 (b). (c) Original image convolved with first-order Gaussian derivative of kernel size *σ* = 5 (c). (d) Original image convolved with first-order Gaussian derivative of kernel size *σ* = 10 (d).

**Figure 3:**
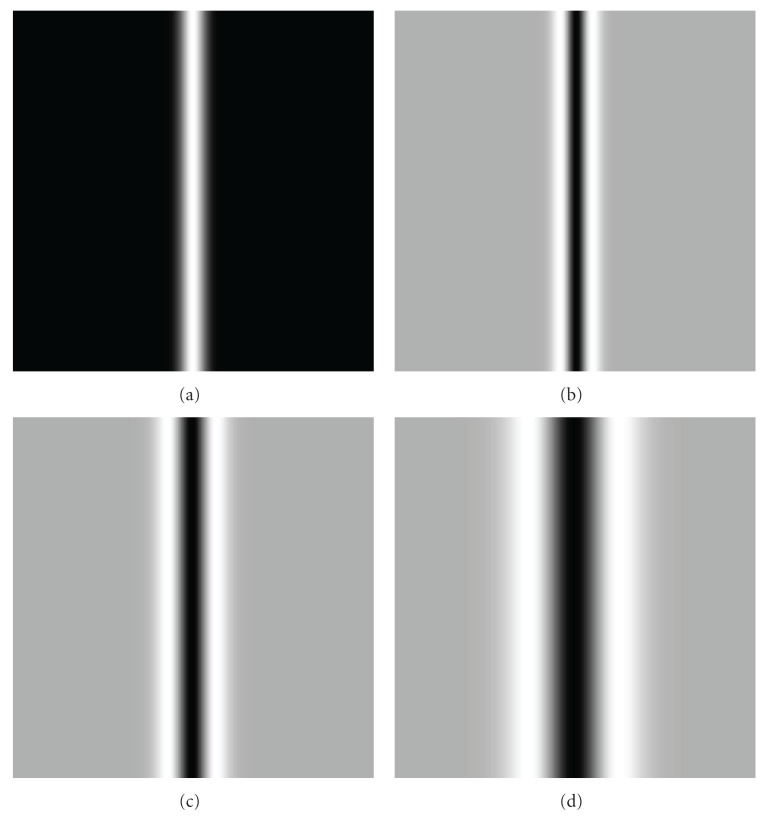
Original image convolved with second-order Gaussian derivatives. (a) Original image has an object with Gaussian intensity profile of kernel size *σ* = 5 (a). (b) Original image convolved with second-order Gaussian derivative of kernel size *σ* = 1 (b). (c) Original image convolved with second-order Gaussian derivative of kernel size *σ* = 5 (c). (d) Original image convolved with second-order Gaussian derivative of kernel size *σ* = 10 (d).

**Figure 4:**
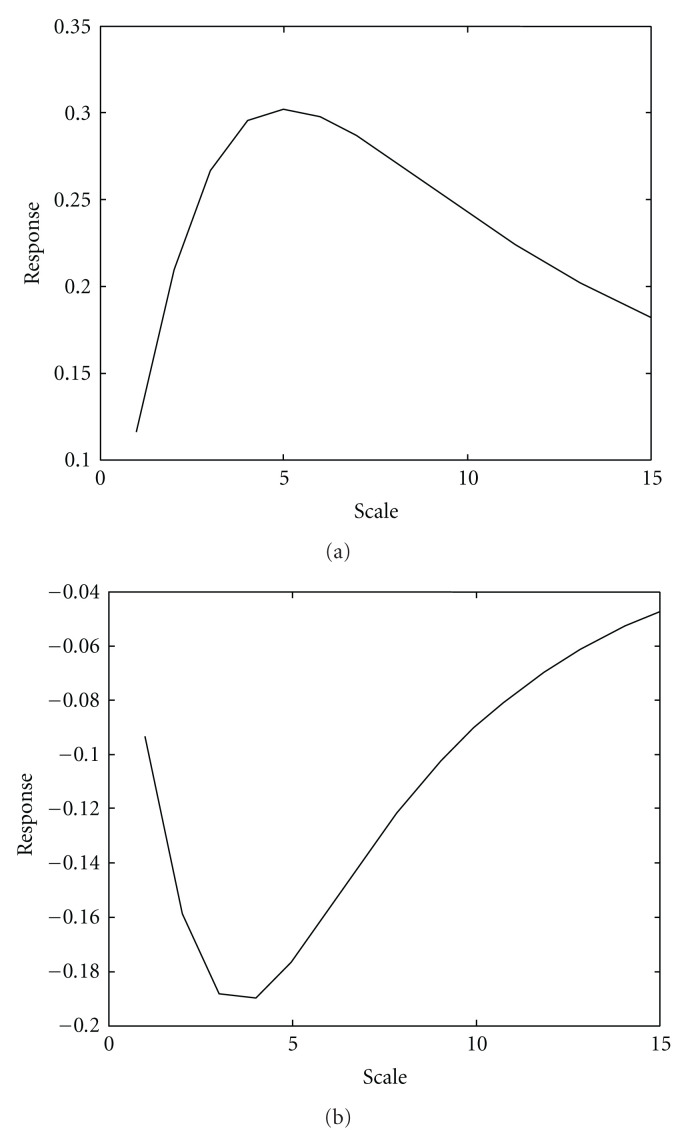
Responses to convolution of the original image with the first- (a) and second- (b) order Gaussian derivatives. Original image has an object with Gaussian intensity profile of kernel size *σ*_obj_ = 5. Gaussian derivative probes scanned over kernel width range *σ*_probe_ = 1–15.

The strongest response of such function (with respect to *σ*) over scales then indicates the proper Gaussian scale probe (Gaussian observation kernel) *σ*_probe_ with width corresponding to object feature size *σ*_obj_ has been found [34]. Due to the commutative properties of the convolution and taking derivative operations, the order of operations in the procedure above could be changed to convolving the original image *I*_0_(*x*, *y*) with the operators constructed from the derivatives of Gaussian (so called “Gaussian derivatives”). The distinguishing characteristic of such operators is a combination of the opposing properties, localization, and optimal response to noise [35–37]. To demonstrate this principle, we modeled the original image with an object with Gaussian intensity profile of kernel size *σ*_obj_ = 5 and convolved this image with the first- (Figure 2) and second- (Figure 3) order Gaussian derivatives with kernel sizes varying over the range *σ*_probe_ = 1–15 (in the figures only *σ*_probe_ = 1, 5, 10 are shown). Then, we measured intensity values of response functions (see (3)), across the object and extracted the strongest responses for all scales of the Gaussian probe. The strongest responses to both first and second Gaussian derivatives with kernel probe varied over the range *σ*_probe_ = 1–15 and convolved with the original image with feature size *σ*_obj_ = 5 are shown in Figure 4. These plots demonstrate the property of the response functions such that it has the strongest response when Gaussian probe size approaches the object size.

Unfortunately, the amplitude of Gaussian derivative operators tends to decrease with increasing scale due to the fact that with increasing scale, the response is increasingly smoothed. This gives more preference to smaller scales. To compensate such increase and, thus, improve accuracy of the automatic scale selection, Lindeberg [10, 32, 34] suggested using the so-called *γ*-parameterized normalized derivatives

(4) $$\frac{\partial^{m+n}}{\partial u^{m}\partial v^{n}}=\sigma^{(m+n)\gamma }\frac{\partial^{m+n}}{\partial x^{m}\partial y^{n}}.$$

This method of scale selection allows feature detectors to find such points in the image that the *γ*-normalized operator response has an extremum with respect to both position and scale.

**Figure 5:**
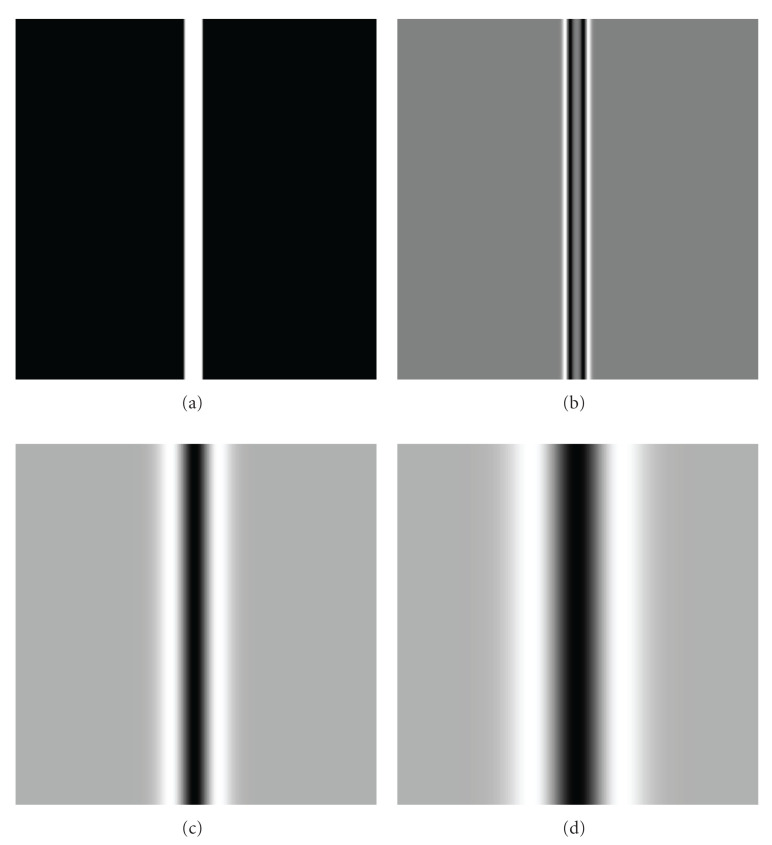
Original image convolved with second-order Gaussian derivatives. (a) Original image having an object with bar-like intensity profile of size 2*σ* = 10 (a). (b) Original image convolved with second-order Gaussian derivative of kernel size *σ* = 1 (b). (c) Original image convolved with second-order Gaussian derivative of kernel size *σ* = 5 (c). (d) Original image convolved with second-order Gaussian derivative of kernel size *σ* = 10 (d).

**Figure 6:**
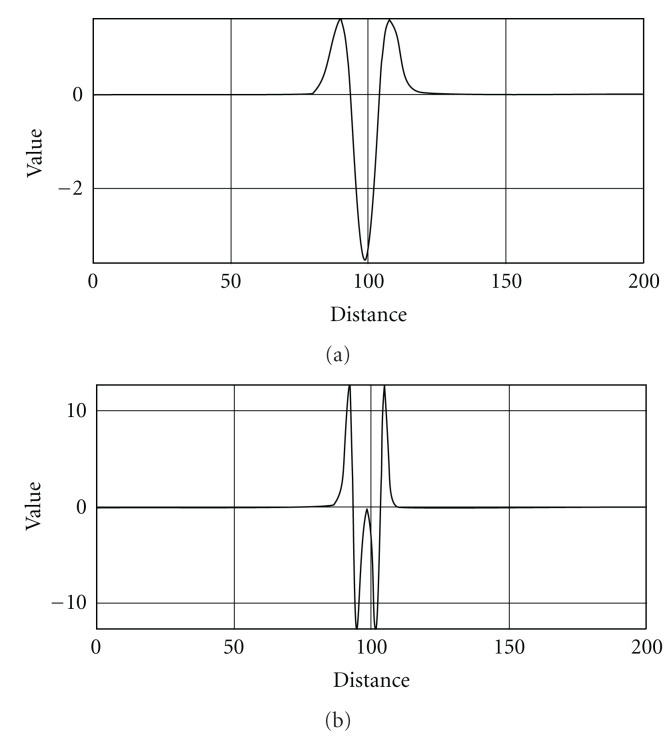
Intensity profiles of responses to Gaussian (a) and bar-like (b) ridge models.

In the case of tubular-like structures in an image, ridge detection with automatic scale selection can be done using a second derivative of Gaussian kernel function [38, 39]. Depending on values of the *γ*-parameter, the detected object features can be quite different. Analyzing the influence of the *γ*-parameter on feature detection with automatic scale selection, Lorenz et al. [40] chose *γ* to be 1.5 which worked well for variety of intensity line profiles (e.g., Gaussian, bar-, triangle-like). However, for elongated structures with bar-like intensity profiles (such intensity profiles can be found in high-quality imagers with narrow point spread functions [41, 42] or imagers that use deconvolution preprocessing algorithms [43, 44]), the ridge detector creates false responses at small scales (basically these are “edge responses at small scales”) as depicted in Figure 5. The line intensity profiles demonstrating the problem are shown in Figure 6. Since in automatic approaches there is no the preferred scale (all scales should be treated equally) in an image, a number of solutions have been proposed to avoid or suppress these false responses in scale space. Koller et al. [45] suggested applying a nonlinear operator that combines the response of two edge-detectors on both sides of a hypothetical ridge. Lorenz et al. [40] used an edge-indicator to suppress the response to edges. Lindeberg [34] used a hybrid approach taking the useful properties from both the scale-space height ridge and the second derivative scale-space ridge. While studying the problem of the influence of the *γ*-parameter on feature detection with automatic scale selection, Majer [38, 39] derived the g-normalization parameter value from the statistical approach based on a white noise sampling model. In these studies, he concluded that the ridges generated by a second-order Gaussian derivative operator do not suffer from the false responses to edges if the value *γ* = 1.25 is used.

Early vision perception occurs at all scale simultaneously and can be modeled by generating the image scale-space representation that introduces an additional variable, spatial scale size [1–4]. At this point, such early vision system is fully ignorant of the geometry. As soon as the local scales are established, the early analysis of an image starts with analysis of intensity variations and directions by means of spatial derivatives to reveal local image structure. For example, for elongated objects, the derivative value along the object is close to zero whereas the derivatives across the object are large negative values and their ratio is close to unity. Following the scale-space ideas developed so far, a complete hierarchical set of scaled differential operators has to be used [25]. The Gaussian derivative operators described earlier in this paper constitute the natural differential operations on a given scale. Thus, a set of Gaussian derivatives and their combinations could be used for very complex object models and analysis. Some well-known combinations of the high-order derivatives have special names like Hessian, Laplacian, and so forth and are used to build special functions for identification of certain shape patterns in images. Such mathematical functions model human and machine perception and are ultimately used in unsupervised object tracking systems [1, 2, 35, 36]. For this purpose, the Hessian-based multiscale object enhancement filters were developed [4, 25, 46–48]. In these anisotropic filters, the pixel intensity transformation is locally governed by the “objectness measure” functions which are built using the combination of local Hessian eigenvalues and calculated in multiscale framework [45, 49–54]. The responses of the filters based on these functions are computed at different scales and are expected to have a maximum value at a scale corresponding to the width of the object [32, 54]. Such selectivity to the object shape along with capability to adaptively choose the optimal scale allows these filters to extract the looked-for objects at their native local scales. As noted by others [32, 55], this is especially important for tubular-like objects axis tracking applications.

The Hessian matrix *H*(*f*) (or simply Hessian) is the square matrix composed of second-order partial derivatives of some scalar-valued multivariable function *f*(*x*_1_, *x*_2_,…, *x*_*n*_), and it describes the local curvatures of this function *f*(*x*_1_, *x*_2_,…, *x*_*n*_). Assuming continuity of the second-order derivatives, the mixed derivatives do not depend on the order of differentiation (e.g., ∂^2^*f*/∂_*x*1_∂_*x*2_ = ∂^2^*f*/∂_*x*2_∂_*x*1_, etc.). The Hessian is then a symmetric matrix which for a 3D image *I*(*x*, *y*, *z*) can be written a 3 × 3 matrix (see (5))

(5) $$H\left(I\right)=\left[\begin{array}{ccc} I_{xx} & I_{xy} & I_{xz}\\ I_{yx} & I_{yy} & I_{yz}\\ I_{zx} & I_{zy} & I_{zz} \end{array}\right].$$

In (5), *I*_*xx*_ = (∂^2^/∂_*x*_∂_*x*_)*I*(*x*, *y*, *z*), *I*_*xy*_ = (∂^2^/∂_*x*_∂_*y*_)*I*(*x*, *y*, *z*), *I*_*xy*_ = *I*_*yx*_, and so on. Due to symmetric property of the Hessian, for 3D images only, six out of nine values have to be calculated. Let the eigenvalues of the Hessian *H*(*I*) be *λ*_1_, *λ*_2_, and *λ*_3_ with their corresponding eigenvectors **e**_1_, **e**_2_, and **e**_3_. If the eigenvalues ordered as *λ*_1_ > *λ*_2_ > *λ*_3_, then the eigenvector **e**_1_ gives the direction of the maximum of the second derivative. 

Following the scale-space ideas described earlier, the partial second derivatives of the image *I*(*x*, *y*, *z*) in the Hessian *H*(*I*) have to be replaced by the *γ*-parameterized normalized Gaussian derivatives (see (4)), convolved with the image which results in that now the eigenvalues *λ*_1_, *λ*_2_, and *λ*_3_ become adjusted to the local size of the tubular object in an image.

Various algorithms for multiscale tubular object tracking and enhancement were developed depending on the way Hessian eigenvalues are combined in the objectness measure function. For instance, Sato et al. [49–51] suggested the “objectness” measure function which used only two out of three values of Hessian eigenvalues

(6) $$\begin{array}{c} f\left(\lambda_{1};\lambda_{c}\right)=\{\begin{array}{cc} \exp\;\left(-\frac{\lambda_{1}^{2}}{2{\left(\alpha_{1}\lambda_{c}\right)}^{2}}\right) & \;\lambda_{1}\leq 0,\;\;\lambda_{c}\neq 0\\ \exp\;\left(-\frac{\lambda_{1}^{2}}{2{\left(\alpha_{2}\lambda_{c}\right)}^{2}}\right) & \;\lambda_{1}>0,\;\;\lambda_{c}\neq 0\\ 0 & \;\lambda_{c}=0. \end{array} \end{array}$$

In (6), *λ*_1_ ≈ 0 and *λ*_2_ ≈ *λ*_3_ ≪ 0 (for a bright line on dark background), *λ*_*c*_ = min (−*λ*_2_, − *λ*_3_), and *α*_1_ < *α*_2_. That filter showed good performance in object enhancement in noisy environment. However, it did not have a parameter to control noise (background) suppression. Frangi et al. [52] extended the “objectness” measure function so that it includes the combination of all three Hessian eigenvalues and a factor with a parameter which controls noise suppression (see more details later in this paper). That function reflects shape and scale of the objects, has a single maximum on the center of the vessels segments, and has bell-shape close to Gaussian with width proportional to object size. This approach showed very good performance and has “de facto” become a basis for building even more sophisticated hybrid filters. Manniesing et al. [53] used this response function to develop an effective denoising filter, where the image intensity transformation is based on anisotropic “diffusion” governed by the “objectness” measure function in multiscale framework. Such an approach, which incorporates the shape pattern analysis along with multiscale data representation, would give us an extremely powerful tool to model artificial system learning. For neuron network reconstruction from 3D confocal microscope images, the tubularity measure function was used to design a statistical learning system for training a classifier and generating the probability that a given structure belongs to the tubular-like object [56, 57]. In pulmonology, the algorithms based on tube detectors were effectively used for airway and lung vascular tree reconstruction from 3D CT images [58, 59]. If properly normalized, the multiscale tube detectors could be used to build various cost and propagation functions required in the level set and fast marching segmentation algorithms [60, 61].

To take the full advantage of the power of the multiscale shape detector filter in object tracking algorithms applied for a large variety of medical applications, in this work we focus on the process of automation of this filter.

The filter itself has many control parameters which can be separated into several groups: Brightness measure (objects are bright relative to background); objectness measure (shape description), scale description (range plus scale step function), and background noise suppression parameter (Frobenius norm scale factor) [52]. Parameters in all groups, except the last one, describe general properties of the object itself so they do not depend on the imaging system characteristics. Since in vascular studies the object brightness, tubularity, and range of diameters are known beforehand, those parameters can be chosen in advance and then fixed. Hence, the only parameter which prevents the algorithm to be fully automated is control of the noise suppression. This parameter depends on the acquisition system and imaging conditions; therefore, it has to be experimentally found for each image set. Such a procedure is very compute expensive.

We present our development of the multiscale Hessian-based tubular object-tracking filter with automatic selection of the parameter used for suppression of background noise. That finalizes the automation of the filter. In our approach, the information required for the parameter calculation is acquired from the image being processed thus it automatically takes into account all the individual properties of the particular image such as voxel size and noise level. This allows for increased automation as well as parallel processing—thereby greatly decreasing processing time. 

## 3. Methods

### 3.1. Images

For our studies, we used both gray-scale images numerically derived and acquired by scanners. The modeled images were programmed so to model environment with certain features. Tubular objects with various widths were placed amid different background: Gaussian random noise, nontubular objects, background with noise, and polynomial varying intensity. For simulations of images degraded by noise, we used the C++ classes contributed to the ITK Insight-Journal by Lehmann [62]. These noise simulation classes are implemented with multithread support and are based on the Mersenne Twister uniform pseudorandom number generator which has a period (2^19937^–1), 632-dimensional equidistribution, and up to 32-bit precision [63]. Thus, this generator could be considered as a “true random” which results in that generated noise does not produce any “neighborhood artifacts” or periodic patterns. The noise was generated with various Standard Deviations SD = 25.0, 50.0, 100.0, and Mean M = 0.0. We also used biomedical images of the heart acquired by the micro-CT scanner [64], Cerebellar Climbing Fibers [65–67], and Hippocampal CA3 Interneuron [67]. Specimen H61 (coronary artery branch within a human heart wall) was a methyl methacrylate cast prepared as described previously [68]. A cast of that coronary arterial tree was scanned with an isotropic voxel size of 0.018 mm and 500 × 500 × 541 voxel CT image volume. 

### 3.2. Micro-CT Scanner

The custom-made micro-CT scanners generate images up to 2048 × 2048 × 1000 isotropic voxels down to 4 *μ*m on a side [64]. 

### 3.3. Server for Image Processing

To be able to process large images using the developed algorithms, we built a specialized server with four 64 bit AMD Opteron 8350 Quad Core 2.0 GHz CPUs and 128 GB memory. The server is located in a server room and it is accessible in multiuser mode through the local network using remote clients. 

### 3.4. Algorithms

For our software development, we used the library of C++ classes from the National Library of Medicine Insight Segmentation and Registration Toolkit (ITK) [69–71]. The library was compiled with multithread support based on the POSIX thread (Pthreads) model [72] using 64 bit C++ compiler GCC 4.3.2 [73–75] and installed on 64 bit Debian Linux 2.6.26 [76, 77]. 

#### 3.4.1. Automation of Multiscale Shape Detector Response Function

The developed multiscale shape detector filter is based on the objectness measure function suggested by Frangi et al. [52] and the C++ classes contributed to the ITK Insight-Journal [78–80]. After thoroughly conducted studies and tests, we found that the C++ classes contributed by Antiga [80] satisfy our purposes the best; therefore, our further developments are based on those classes.

Let *λ*_*k*_ be the eigenvalue of the Hessian matrix at voxel *x* ordered such that |*λ*_1_ | ≤|*λ*_2_ | ≤|*λ*_3_| (we drop the dependency to *x*). In the case of the ideal bright tubular structure, the voxels should satisfy the following relation for eigenvalues |*λ*_1_ | ≈ 0; |*λ*_1_ | ≪|*λ*_2_|; *λ*_2_ ≈ *λ*_3_ and for bright objects both *λ*_2_ and *λ*_3_ must be negative.

Frangi et al. [52] proposed to use the eigenvalues to define Vesselness measure *ν*(*x*) as below:

(7) $$\nu \left(x\right)=\{\begin{array}{c} 0\;\text{if}\;\lambda_{2}>0\;\text{or}\;\lambda_{3}>0,\\ \left(1-\exp\;\left(-\frac{R_{a}^{2}}{2a^{2}}\right)\right)\exp\;\;\left(-\frac{R_{b}^{2}}{2b^{2}}\right)\left(1-\exp\;\left(-\frac{S^{2}}{2c^{2}}\right)\right), \end{array}$$

where *a*, *b*, and *c* are the parameters that control the sensitivity of the filter to the measures *R*_*a*_, *R*_*b*_, and *S*. The measures have the following meaning. *R*_*a*_ = |*λ*_2_ | /|*λ*_3_| is used to distinguish between plate-like and line-like patterns. *R*_*b*_ = |*λ*_1_ | /sqrt(|*λ*_2_*λ*_3_|) is used to derive a blob-like pattern. The measures *R*_*a*_ and *R*_*b*_ are gray intensity level invariant and capture only the topological information of the objects in the image. The choice of the control parameters *a* and *b* defines the object pattern to be studied. For example, if tubular-like objects are chosen, the parameters are fixed to *a* = 0.5, *b* = 0.5 [54].

The parameter *c* (see (7)), controls background noise suppression in the Hessian-based object enhancement filter. The Frobenius Hessian matrix norm is chosen as a measure *S* = sqrt(*λ*_1_^2^ + *λ*_2_^2^ +  *λ*_3_^2^) to distinguish background noisy pixels. Since the parameter *c* strongly depends on the individual properties of particular image such as voxel size and noise level, for every new study, the optimal parameter value should be experimentally found again by trial. The very wide search range of the optimal parameter value in concert with a highly time-expensive calculation to derive the Hessian taken in multiscale framework makes this algorithm very labor-inefficient especially for large 3D biomedical images with high resolution. For example, one trial run to process the 16 bit 500 × 500 × 514 gray-scale micro-CT image on our server took about an hour; thus, the interactive search for the parameter *c* might take hours for the user who operates the program interactively.

The method for automating the selection of the parameter for suppression of background noise uses a scalar function (nondirectional) of the image voxels the Laplacian of the image. The Laplacian is a well known operator in image processing which is easy to calculate [81–83]. Ultimately, the Laplacian calculates the trace of Hessian matrix or, equivalently, the sum of its eigenvalues (*λ*_1_ + *λ*_2_ + *λ*_3_) making it invariant with respect to a change of tensor basis. This characterization can be used to steer the control parameter responsible for noise suppression. The schematic description of complete algorithm is below.

1. For each voxel in the image, calculate the Laplacian.
2. In the calculated Laplacian array, find the maximum value of Laplacian, that is, (*λ*_1_+*λ*_2_+*λ*_3_)_max_.
3. Take one tenth of that maximum value of Laplacian, that is, (*λ*_1_+*λ*_2_+*λ*_3_)_max _/10.
4. Assign the calculated value to parameter *c*.

In this approach, the information required for parameter calculation is acquired solely from the image being processed; thus, it automatically takes into account all the individual properties of the particular image such as a voxel size and noise level. 

#### 3.4.2. Objective Measures for Perceptual Quality Evaluation of Images

There are two measures commonly used for objective evaluation of the perceptual quality of images: Mean Square Error (MSE) and Peak Signal to Noise Ratio (PSNR) [84–90]. These quality measures are defined as follows. Consider two images being compared **x** = {*x*_*i*_ | *i* = 1,2,…, *N*} and **y** = {*y*_*i*_ | *i* = 1,2,…, *N*}, where *N* is the number of points (pixels) in the data sets and *x*_*i*_ and *y*_*i*_ are intensity levels in the images. Let **x** be an “ideal image” and **y** be a “degraded image”. The MSE measure is then defined as MSE = (1/*N*)∑_*i*=1_^*N*^(*x*_*i*_ − *y*_*i*_)^2^ and PSNR is, respectively, PSNR = 10 log_10_(*L*^2^/MSE) where *L* is the dynamic range of allowable pixel intensities. As follows from the definitions above, the lower the values of MSE, the lower the error and the higher the PSNR, the better quality of processed image.

The MSE and PSNR measures were developed using C++ classes from the ITK library [69] and contribution [62]. 

## 4. Results

### 4.1. Manual Mode

**Figure 7:**
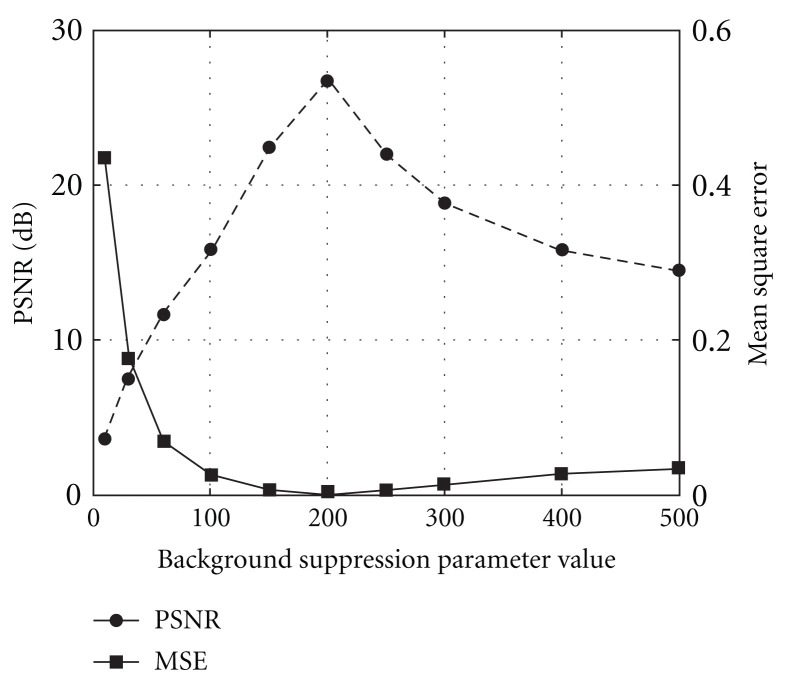
Mean Square Error (MSE) and Peak Signal-to-Noise Ratio (PSNR) measurements for manual image processing.

In manual mode, the control parameters have to be provided by the operator before running the code. If the result is not acceptable, the operator has to change parameters and rerun the code again. The MSE and PSNR measures were used for objective quality evaluation of the processed image. As an “ideal image”, there was used a modeled image comprised three tube-like objects with Gaussian intensity profiles of different width. Then, the ideal image was degraded by the random Gaussian noise with Standard Deviation SD = 100.0 and Mean M = 0.0 and processed in manual mode by the algorithm. The background suppression parameter value was sampled over a wide range 10–500 to surely cover prospective optimal control parameter. The results are depicted in Figure 7. It can be seen that both measures indicate the optimal value is about *c* = 200 which corresponds to MSE value 0.00214 and PSNR value 26.69. If run in automatic mode, the algorithm finds even more precise the optimal value *c* = 194.3 which corresponds to MSE value 0.00203 and PSNR value 26.93. These results demonstrate capability of the developed algorithm in automatically finding the optimal control parameter for background suppression. 

### 4.2. Fully Automatic Mode

To test efficiency of the algorithm in fully automatic mode, we used the modeled images described above. The control parameters were chosen and fixed: brightness “on”, objectness measure “tubular”, scale range “1–30”, scale steps “20”, step function “logarithmic”, noise suppression mode “automatic”. Scale range was chosen wide enough to cover all possible diameters. The step function was made “logarithmic” so as to emphasize finer scales. 

#### 4.2.1. Background with Gaussian Random Noise and Nontubular Objects

**Figure 8:**
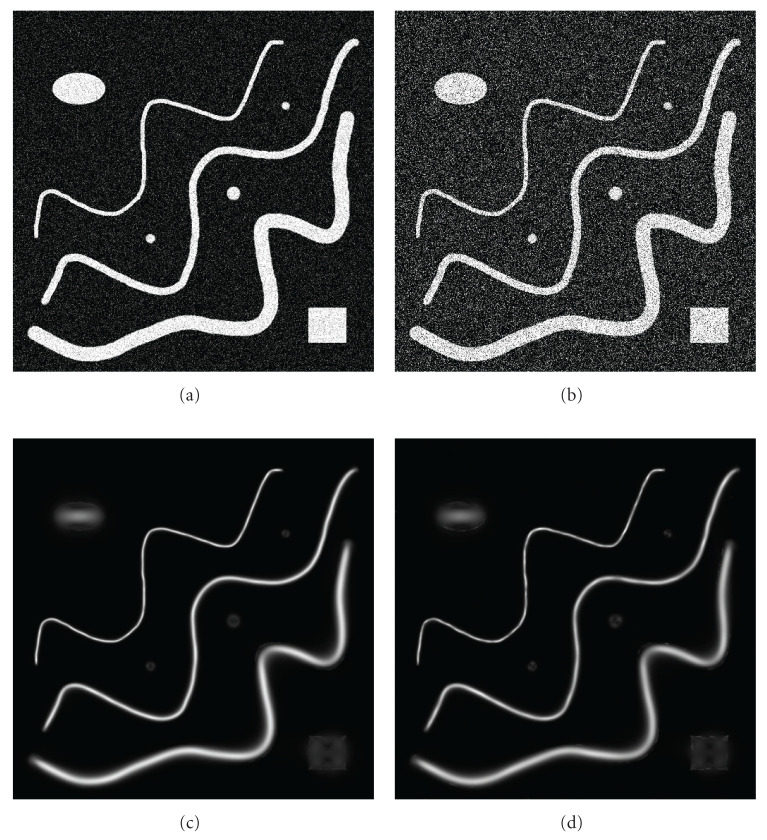
Automatic processing. Input images with noise mean = 0 and standard deviation (a) SD = 25 and (b) SD = 50; processed images (c) and (d).

First, we processed the images with curved tubular objects with various widths which were placed amid nontubular objects. The images were degraded by Gaussian random noise with SD = 25.0, 50.0, and M = 0.0. As can be seen from Figure 8, the algorithm automatically finds the optimal parameters and successfully tracks tubular objects. 

#### 4.2.2. Objects in Images with Nonlinear Background

**Figure 9:**
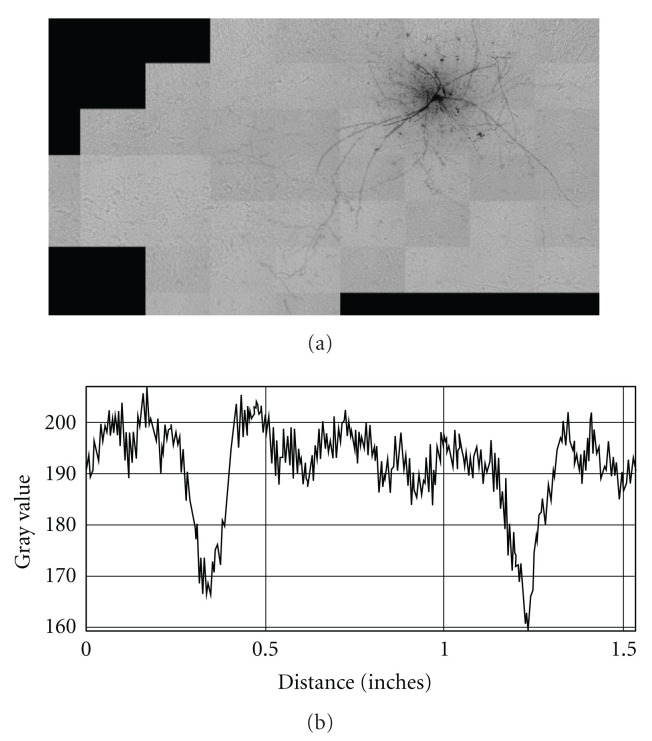
Neurological confocal microscopy image of Hippocampal CA3 Interneuron with polynomial noisy background: (Image data courtesy from Professor German Barrionuevo).

**Figure 10:**
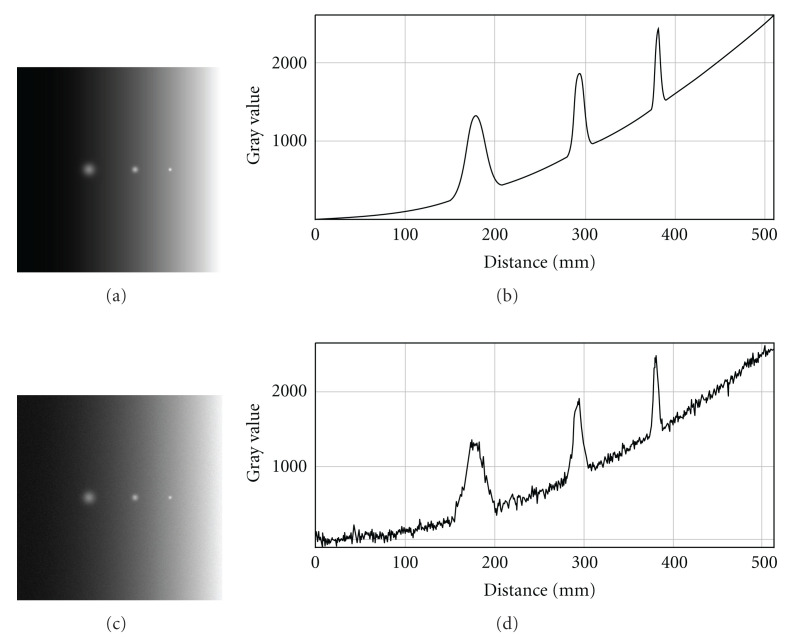
Program-simulated images with polynomial noisy background and tubular objects with Gaussian intensity profiles.

**Figure 11:**
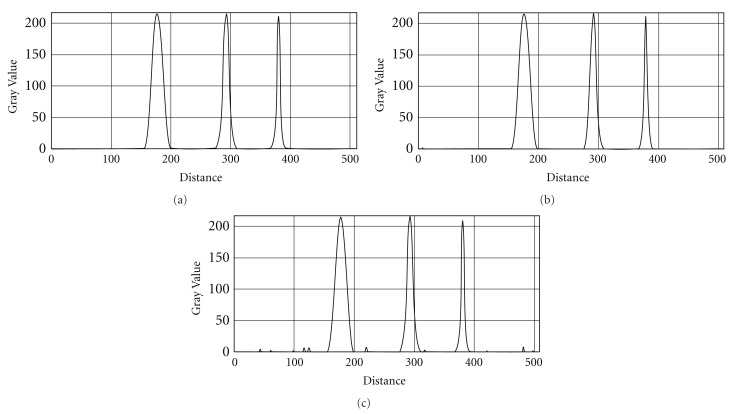
Intensity profiles across the tubular objects with Gaussian intensity profiles in the processed images with various polynomial noisy backgrounds added. (a) Noise SD = 0; (b) SD = 25; (c) SD = 50.

We also tested performance of the algorithm for neurological confocal microscopy image processing affected by tiling, shading, Gaussian noise, nonlinear background, and so forth (see, e.g., Figure 9). The modeled images were programmed so to present three parallel tubular objects aligned in the Z-direction with different diameters and distance between objects along with gray-scale distribution described by Gaussian intensity profiles. The polynomial background was added with and without Gaussian random noise (Figure 10). In Figure 11, there are depicted intensity profiles across the tubular objects with Gaussian intensity profiles in the processed images with various polynomial noisy backgrounds added SD = 0.0, 25.0, 50.0, and Mean M = 0.0. As could be seen, the algorithm effectively suppresses the background and successfully extracts the tubular images. 

### 4.3. Speed Efficiency of Fully Automatic Mode

To evaluate time efficiency of our automatic method, we processed the 500 × 500 × 514 (16 bits per voxel) micro-CT image with 0.018 cubic mm size in multithreaded mode using a Linux-based server with the 16 processors and 128 GB memory as described above. First, we ran the automatic algorithm to find the parameter *c*. Then, we ran nonautomatic (the found parameter was manually plugged into a program) version. The amount of CPU time spent for a single run in non-automatic mode is 58 minutes whereas for automatic mode is 64 minutes, which is just about 10% longer. If the server is able to allocate enough memory for the run, the time spent by the algorithm in fully automated mode is comparable with time for the nonautomatic mode. 

### 4.4. Biomedical Images

**Figure 12:**
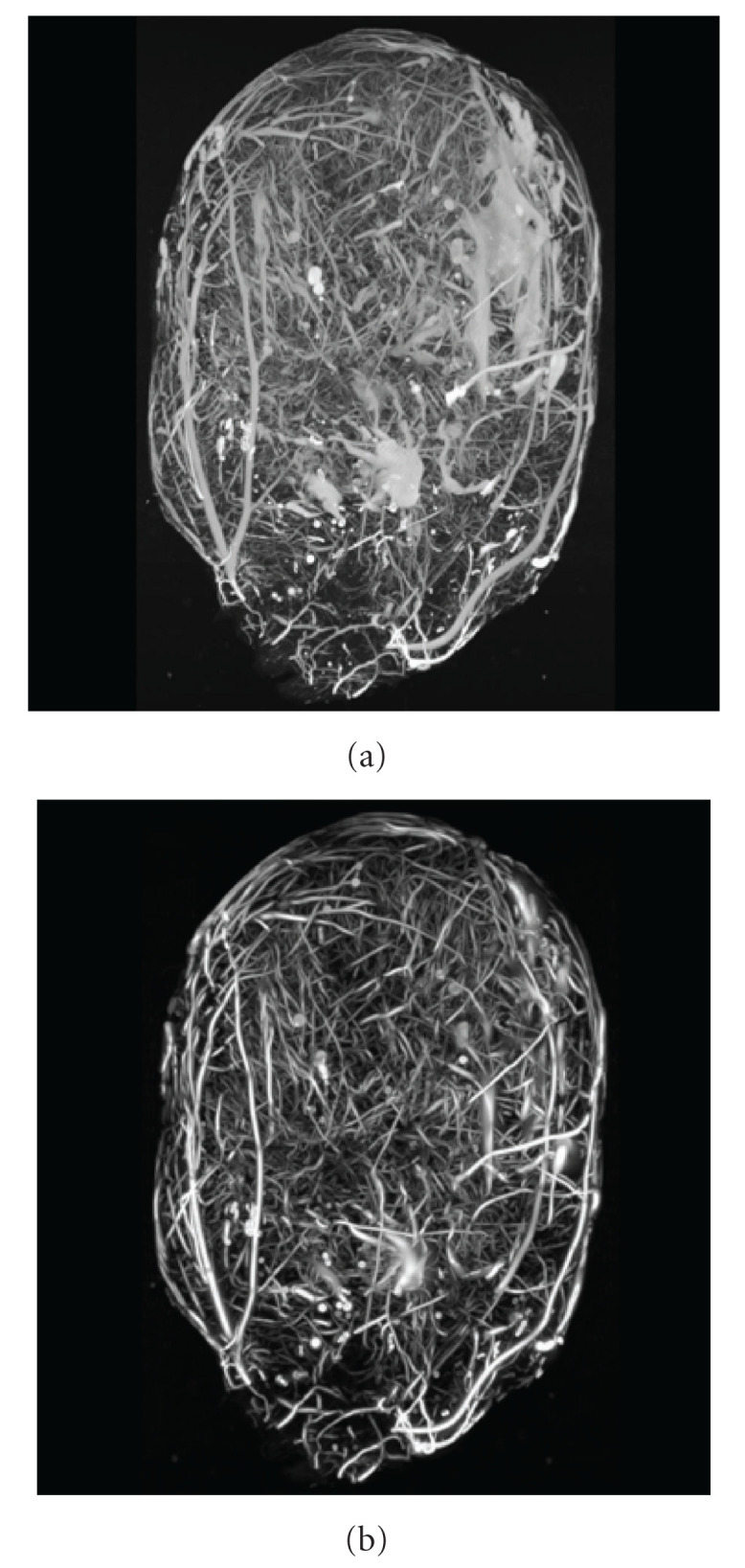
Maximum Intensity Projection (MIP) of micro-CT image of rat heart coronary arteries. (a) Input image; (b) Processed image.

The result of applying our automatic algorithm to the micro-CT image of the coronary arteries in a heart in concert with the nonprocessed image is shown in Figure 12 using Maximum Intensity Projection (MIP) images. Before preprocessing (a) there are the “blobs” of white material due to a contrast medium accumulation in a cardiac chamber. Those “blobs” are selectively removed by the algorithm as can be seen in right panel (postprocessing).

**Figure 13:**
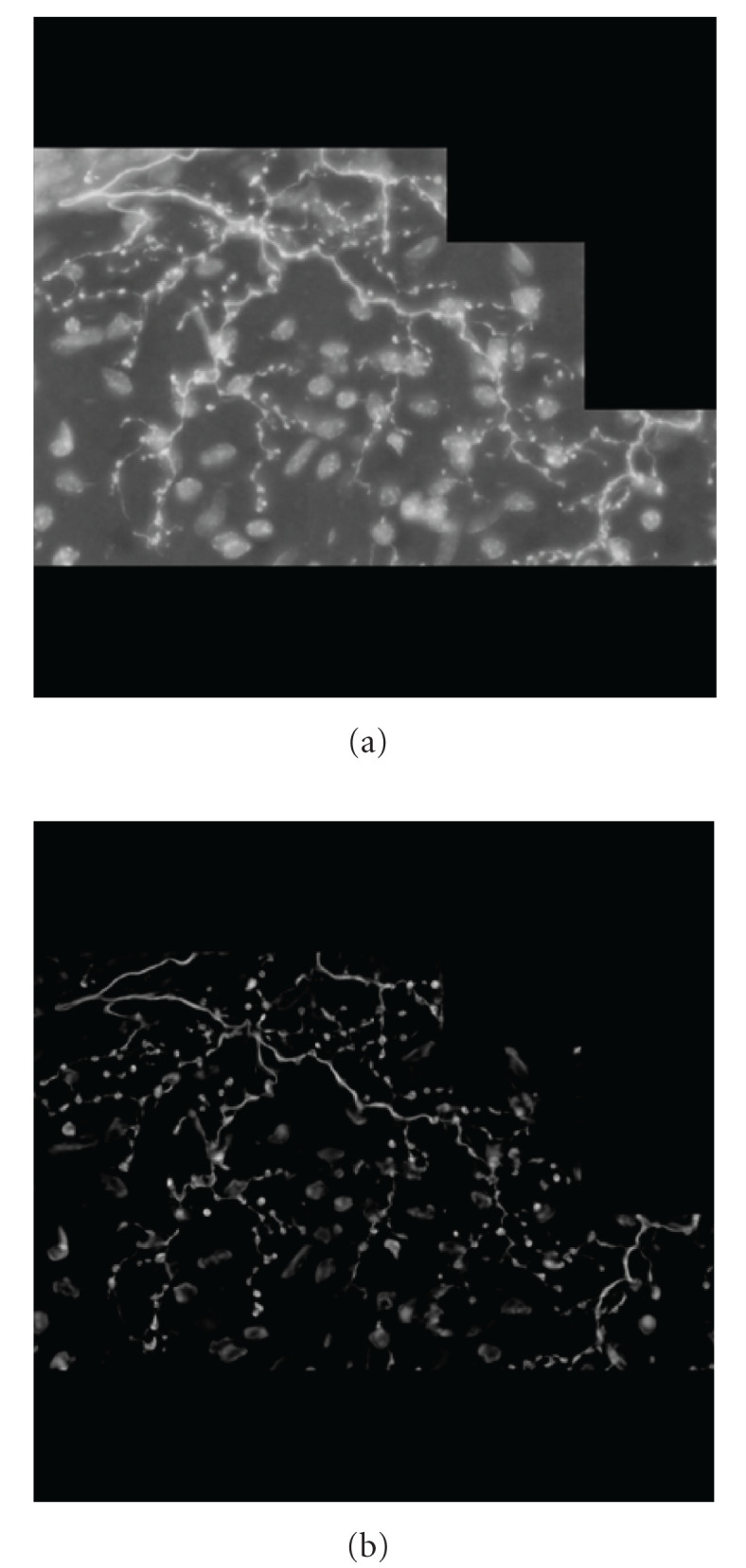
Histological image of cerebellar climbing fibers. (a) Input image; (b) Processed image (Image data courtesy from Professor Giorgio Ascoli).

In Figure 13, there are the not processed (a) and processed (b) MIP images of cerebellar climbing fibers. As expected, the tubular measure filter effectively suppresses the fixed pattern background and noise and “delineates” the tubular objects. 

**Figure 14:**
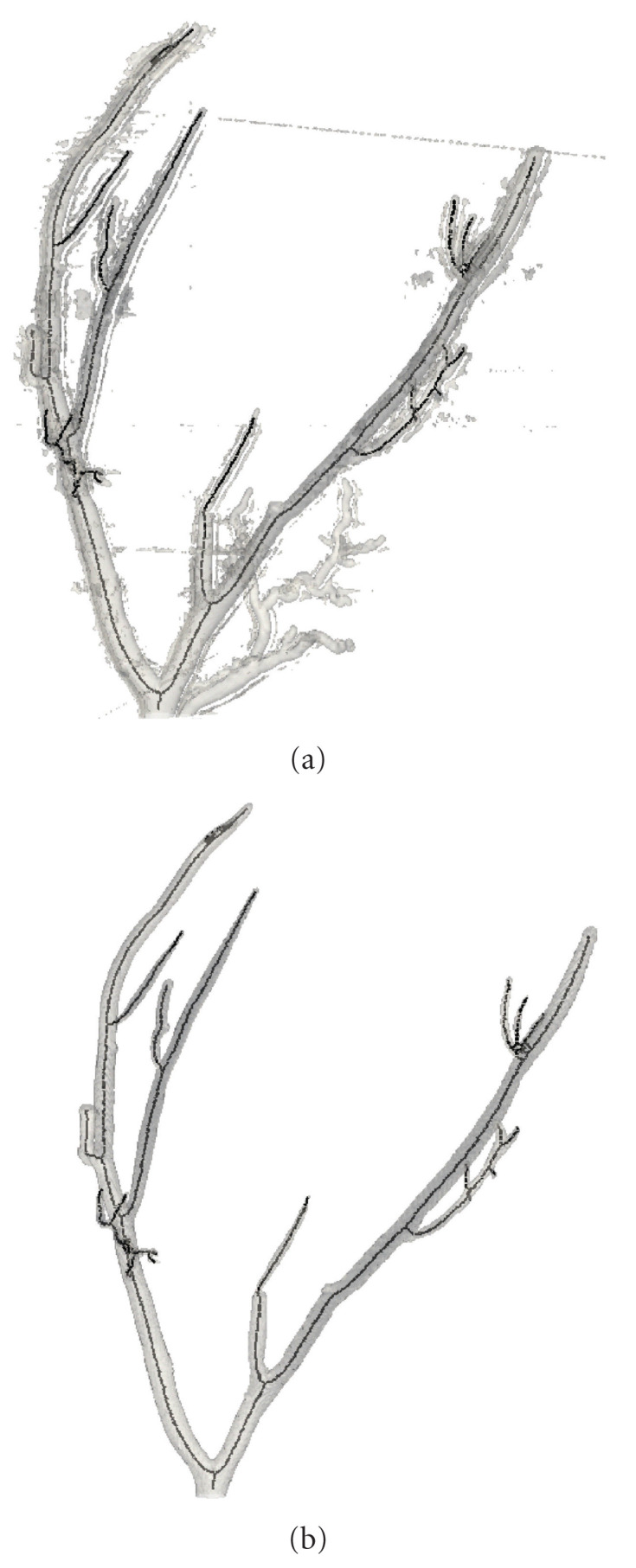
Specimen H61 (coronary artery branch within a human heart wall). (a) Not processed image with extracted center line; (b) Segmented processed image with extracted center line.

We also explored the efficiency of the algorithm as an initial filter in the central line extraction pipe line. The original image Sample H61 was processed with the developed filter and then segmented using the region growing connected threshold algorithm [69–71]. The seed for segmentation was allocated at the beginning of the main tree root thus the side trees were excluded. Afterwards, a center line was extracted using the 3D thinning approach based on the C++ classes submitted to the insight journal by Homann [91]. Images along with the extracted center lines are depicted in Figure 14. As could be seen from the figure, the algorithm effectively suppresses background and delineates the tree. The centerlines are extracted correctly as well. 

## 5. Discussion and Future Work

We have presented a method for automation of adaptive nonsupervised system for tracking tubular objects that is based on analysis of local structures performed in multiscale framework. The designed filter has demonstrated a great potential for complete automation and showed very good performance in both background noise suppression and tubular object tracking. 

The developed approach can be used in the reconstruction pipeline right after image deconvolution operation. Even though the convolution operator will reconstruct the object features at finer scales, those features will appear in increased noise environment which in return might require additional postprocessing for noise suppression yet to preserve extracted features.

Another application is the object feature extraction pipeline. This filter can be used as a preprocessing filter for vessel enhancement and background noise suppression right before segmentation or immediately in the segmentation algorithms itself, for instance, in the family of segmentation algorithms which require distributed seeds [70, 71]. By using the thresholded output of the vessel enhancement filter as a seeder, it can increase the speed efficiency of the segmentation process considerably.

Since the response function is built using exponents, with proper normalization this function can be considered as a probability function with values distributed over the interval “0.0-1.0”. In this case, after processing, the output image holds voxels with values of probability of the event that “a voxel belongs to the object with tubular shape”. These probabilities can be used in many ways. The most traditional way is to rescale it back to a gray-scale image. Although such images do not keep a proper intensity calibration, they still can be used for morphometric analysis. If calibration is of concern, the probabilities could be converted to a mask for sampling the original micro-CT image from which the calibration could be recovered. 

Since the filter generates the response function with only one maximum across scale space at a scale that is proportional to the diameter of the tubular object and that maximum is located at the center of the object, the probability image is more suitable to construct various cost functions. The images with cost functions can further be used as the “feature image” in various image processing pipelines, for instance, such as in flux-driven centerline extraction algorithms [92, 93], level-set and fast-marching segmentation algorithms [61, 69–71], and so forth. As the multiscale vessel enhancement filter is very robust against noise, it can be superior over traditional approaches like, for example, in a filter pipeline “segmented image, distance map, and cost function”, since it can directly generate the cost function avoiding steps for producing segmentation and distance map. In addition, the multiscale space feature can be used to build the cost function in multiscale representation and use it for multiscale vessel tracking as suggested in [54].
